# Offer of a menu of different nicotine substitute products to REduce Tobacco Use iN pEople living with HIV (RETUNE): a protocol for a pragmatic randomized trial within the Swiss HIV Cohort Study

**DOI:** 10.1186/s13063-026-09622-6

**Published:** 2026-04-08

**Authors:** Christof Manuel Schönenberger, Benjamin Speich, Elias R. Zehnder, David Hans-Ulrich Haerry, Ellen Cart-Richter, David Jackson-Perry, Alissa Hutter, Samuel Aggeler, Julian Steinmann, Sandra E. Chaudron, Alexandra Calmy, Matthias Cavassini, Dominique Braun, Christoph A. Fux, Irene Abela, Katharina Kusejko, Huldrych F. Günthard, Johannes Nemeth, Andri Rauch, Gilles Wandeler, Patrick Schmid, Tamara Dörr, Julia Notter, Maja Weisser Rohacek, Bernard Surial, Aurélie Berthet, Reto Auer, Marcel P. Stoeckle, Niklaus Labhardt, Frédérique Chammartin, Matthias Briel, Alain Amstutz, the Swiss HIV Cohort Study

**Affiliations:** 1 Division of Clinical Epidemiology, Department of Clinical Research, University Hospital Basel and University of Basel, Totengässlein 3, Basel, 4051 Switzerland; 2 RETUNE-HIV Participant Advisory Board and President of the patient organization “Positive Council”, Bern, Switzerland; 3 RETUNE-HIV Participant Advisory Board and Lausanne HIV-Community Council, University Hospital Lausanne, University of Lausanne, Lausanne, Switzerland; 4 RETUNE-HIV Participant Advisory Board and Infectious Diseases Service, University Hospital Lausanne, University of Lausanne, Lausanne, Switzerland; 5 Division of Infectious Diseases and Hospital Epidemiology, University Hospital Zurich, Zurich, Switzerland; 6 Institute of Medical Virology, University of Zurich, Zurich, Switzerland; 7 Division of Infectious Diseases, University Hospital Geneva, University of Geneva, Geneva, Switzerland; 8 Division of Infectious Diseases, University Hospital Lausanne, University of Lausanne, Lausanne, Switzerland; 9 Hirslanden Klinik Zurich, Zurich, Switzerland; 10 Division of Infectious Diseases, Cantonal Hospital of Aarau, Aarau, Switzerland; 11 Department of Infectious Diseases, Inselspital, Bern University Hospital, University of Bern, Bern, Switzerland; 12 Division of Infectious Diseases, Infection Prevention and Travel Medicine, HOCH Health Ostschweiz, Cantonal Hospital St. Gallen (University Teaching and Research Hospital), St. Gallen, Switzerland; 13 Division of Infectious Diseases, University Hospital Basel, University of Basel, Basel, Switzerland; 14 Center for Primary Care and Public Health (Unisanté), University of Lausanne, Lausanne, Switzerland; 15 Institute of Primary Health Care (BIHAM), University of Bern, Bern, Switzerland; 16 Department of Health Research Methods, Evidence, and Impact, McMaster University, Hamilton, Canada; 17 Bristol Medical School, Population Health Sciences, University of Bristol, Bristol, UK; 18 Oslo Centre for Biostatistics and Epidemiology, Oslo University Hospital, University of Oslo, Oslo, Norway

**Keywords:** TwiCs, HIV, Smoking, Nicotine

## Abstract

**Background:**

Among people living with HIV, there has been a shift of focus from HIV-related health issues to cardiovascular diseases and cancer. For both, tobacco smoking is a major but insufficiently addressed etiological factor. Evidence from randomized trials suggests that nicotine substitute products such as e-cigarettes and nicotine patches can reduce tobacco smoking and its associated health burden. However, most previous smoking cessation trials primarily included people who are motivated to quit smoking and focused on testing a single nicotine substitute product. The effectiveness of offering a menu of nicotine substitute products to tobacco smokers regardless of their willingness to quit smoking (“opt-out” approach) is unknown.

**Methods:**

*Reduce tobacco use in people living with HIV in Switzerland* (RETUNE, NCT06789692) is a pragmatic, 1:1 randomized, multicenter, superiority clinical trial using the Trials within Cohorts (TwiCs) design within the Swiss HIV Cohort Study. RETUNE assesses the effectiveness of offering a menu of different nicotine substitute products, namely electronic cigarettes, nicotine pouches, and nicotine patches, versus usual care. Cohort participants are eligible if they smoke more than one tobacco cigarette per day, do not use any of the substitute products, and have signed the randomization consent following the TwiCs design. Participants randomized to the intervention may choose any of the offered substitutes to be used free of charge for 6 months or decline the offer. Overall, we plan to recruit 972 participants. The primary outcome is tobacco abstinence at 6 months measured as participant-reported past 7-day prevalence abstinence. The primary outcome will be assessed in the intention-to-treat set using a logistic regression model adjusted for region, men having sex with men, current drug users, and number of cigarettes per day at baseline. Secondary outcomes are long-term smoking cessation rates and tobacco-associated health outcomes.

**Discussion:**

RETUNE started recruitment in February 2025 and is currently ongoing. RETUNE using the TwiCs design will clarify the effectiveness of a preference-based opt-out smoking cessation intervention among people living with HIV.

**Trial registration:**

Clinicaltrials.gov NCT06789692. Registered on January 17th, 2025. The manuscript is aligned with the registry. https://clinicaltrials.gov/study/NCT06789692?cond=NCT06789692&rank=1

**Supplementary Information:**

The online version contains supplementary material available at 10.1186/s13063-026-09622-6.

## Introduction

People living with HIV (PLWH) have a close-to-normal life expectancy due to highly effective antiretroviral therapy [1]. Among PLWH, there has been a shift of focus from HIV-related health issues to non-communicable diseases, especially cardiovascular diseases [1–4] and cancer [5–7]. Two meta-analyses suggested a doubled overall risk for cardiovascular disease in this population compared to the general population [2, 8, 9]. Moreover, the incidence of lung cancer is higher in PLWH [10, 11]. Tobacco smoking is a key contributor for both cardiovascular diseases and cancer, thus has major health implications among PLWH [5, 12]. Currently, smokers with HIV lose more life years to smoking than to HIV [5].

There is widespread consensus that nicotine, while addictive, is not responsible for the development of cardiovascular diseases or cancer [13, 14]. Therefore, harm reduction interventions using nicotine substitute products have become more popular in smoking cessation programs. Conventional nicotine replacement therapy, such as transdermal patches, is well established and effective [15–17]. However, insufficient imitation of pharmacodynamic and behavioral properties of cigarette smoking may limit their effectiveness [16]. In contrast, electronic cigarettes (e-cigarettes) come closer to the smoking routine and mimic experiences of smoking by faster nicotine absorption rate and therefore improve cessation rates compared to conventional nicotine replacement therapy [18, 19]. Although there is still a lack of long-term data, current evidence suggests that e-cigarettes are notably less harmful than tobacco cigarettes with respect to the development of cancer and lung disease [20]. Tobacco-free nicotine pouches are novel nicotine products for oral use and also imitate smoking better than traditional nicotine replacement therapy [21, 22]. So far, no randomized trial has investigated the effectiveness of nicotine pouches to quit tobacco smoking. Nevertheless, the FDA has officially added nicotine pouches as a harm reduction tool for tobacco smoking [23].

Most smoking cessation trials require participants to be willing to quit smoking within a certain time window after inclusion (“opt-in” approach) which limits applicability of results in clinical routine [24–28]. It has been suggested that smokers who want to quit and smokers without this explicit intention have similar quit rates, provided they receive appropriate support [29–31]. However, evidence from randomized clinical trials on the effectiveness of an “opt-out” approach is conflicting [32, 33]. An US-based trial by Richter and colleagues investigated a complex intervention containing counselling and smoking cessation medication (nicotine patches, gums, lozenges) among 1000 participants but was unable to show increased cessation rates [32]. Another trial conducted by Carpenter and colleagues in the US showed high acceptance and effectiveness of offering e-cigarettes using an “opt-out” approach. The e-cigarette use among 638 participants was 70%, and the self-reported point prevalence abstinence in the intervention was higher after 6 months than in the control group (odds ratio 1.8, 95% confidence interval 1.0–3.1) [33]. Whether offering a menu of different nicotine substitute products can improve uptake and cessation rates in an “opt-out” setting is unclear. A pilot study indicates high acceptance of e-cigarettes as offered as a nicotine substitute product [34], but evidence from randomized trials is rare [35].

The *Reduce Tobacco Use in People living with HIV in Switzerland* (RETUNE) trial will investigate the effectiveness of offering a menu of different nicotine substitute products, including e-cigarettes, nicotine pouches, and nicotine patches, to all smokers within the Swiss HIV Cohort Study (SHCS), regardless of their willingness to quit smoking, using the Trials-within-Cohorts (TwiCs) design.

## Methods

### Setting

The SHCS, established in 1988, is a multicenter, prospective, observational, nationwide cohort study [36]. PLWH are recruited at all university hospitals in Switzerland and at various other clinics, and regular follow-up visits are conducted every 6 months. The cohort currently includes about 9500 active participants [37]. In June 2024, the SHCS amended their protocol to enable the conduct of trials using the TwiCs design. RETUNE will recruit participants at the SHCS centers in Basel, Bern, Geneva, Lausanne, St. Gallen, Zurich, and Aarau.

### Patient and public involvement

All phases of the project so far (planning phase, acquisition of funding, intervention design, informed consent forms, protocol writing, writing of this manuscript) were supported by the RETUNE participant advisory board. The members of the participant advisory board are co-authors of this manuscript. We will use the Guidance for Reporting Involvement of Patients and the Public (GRIPP-2) checklist to report the patient and public involvement in the publications of the trial results [38].

### Design

**Fig. 1:**
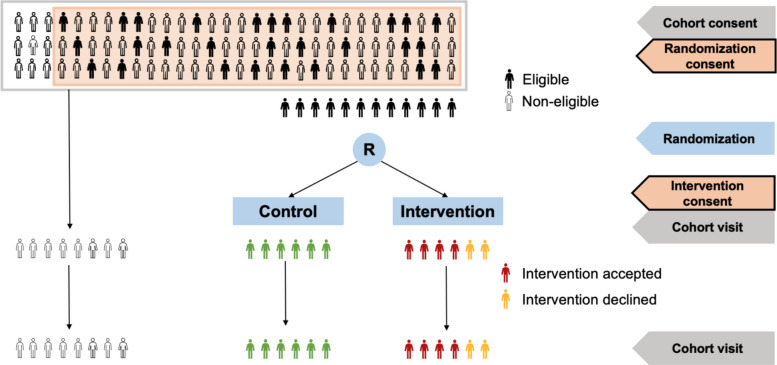
Trials Within Cohorts (TwiCs) design. To be part of the SHCS, all participants need to sign the “cohort consent.” Since the amendment of the SHCS protocol in August 2024, participants are also routinely asked for an additional “randomization consent.” Smokers who signed the “randomization consent” and without exclusion criteria become eligible for the RETUNE trial. They are randomized in a 1:1 ratio. Participants in the intervention group may accept or decline the offered intervention. If they accept the intervention, they sign an “intervention consent.” The primary analysis will be conducted using an intention-to-treat analysis set. All trial endpoints are collected within the routine cohort visits

RETUNE is a multicenter, pragmatic, 1:1 randomized, superiority clinical trial using the TwiCs design. In the TwiCs design, participants are recruited within a prospective cohort study (see Fig. 1). Cohort participants give their consent not only to regular data collection at cohort visits (cohort consent), they are also asked for their consent to be randomized into future trials nested in the cohort (randomization consent) [39, 40]. Eligible participants for such a trial are then randomized to intervention and control. If they are randomized to the intervention, they are informed, can accept or decline the offered intervention and sign an additional consent (intervention consent). Participants randomized to the control arm are not informed about the trial but followed continuously in the frame of the cohort and their data serve as the control. In TwiCs, the control group receives usual care following the cohort standards. Importantly, all trial outcome data are collected within the routine cohort data collection. We used the *Standard Protocol Items: Recommendations for Interventional Trials (SPIRIT)* guideline for this report (the completed checklist is available as supplement 1) [41].

Participants in the intervention group can withdraw from the intervention at any time. However, they remain part of the SHCS and its data collection unless they also withdraw cohort participation.

### Primary hypothesis and primary estimand

We hypothesize that offering a preference-based smoking cessation intervention menu, consisting of e-cigarettes, nicotine pouches, and nicotine patches, in addition to usual care, is safe and superior to reduce tobacco smoking among PLWH in the SHCS after 6 months compared with usual care alone. The primary estimand [42, 43] and the handling of intercurrent events are described in Table 1.

**Table 1:** Description of the primary estimand and handling of intercurrent events

Attribute	Definition
Population	Adults living with HIV in Switzerland, smoking one or more tobacco cigarettes per day
Treatment condition	
Intervention	Offer of different nicotine substitute products (consisting of e-cigarettes, nicotine pouches and nicotine patches) free of charge for 6 months
Control	Usual smoking cessation care in the SHCS
Endpoint	Self-reported abstinence from tobacco smoking in the past 7 days at 6 months
Summary measure	Odds ratio between groups, adjusted for key prognostic baseline covariates
Handling of intercurrent events	
Death (truncating event)	While-alive strategy: Only outcomes before the occurrence of the death are considered
Non-uptake of any smoking cessation product in the intervention group and uptake of an intervention smoking cessation product in the control group (treatment-modifying event)	Treatment policy strategy: The occurrence of these intercurrent events is part of the treatment condition. Participants stay in the intention-to-treat analysis set as randomized at baseline regardless of (non-)uptake thereafter
Stop or switch of use of a smoking cessation product at any time during the intervention due to any reason (treatment-modifying event)	Treatment policy strategy: The occurrence of the intercurrent event is part of the treatment condition. Participants stay in the intention-to-treat analysis set, regardless of their adherence to the intervention
Attrition from SHCS (truncating event)	Principal stratum strategy: Participants dropping out of the SHCS are excluded from the analysis set, assuming missing at random (no common cause between outcome and attrition), occurring at similar frequency across both groups and considered as such in the power calculation

### Screening, consent procedure, and eligibility

During routine cohort visits, the treating physicians collect follow-up data using the SHCS data collection tool. Simultaneously, cohort participants are assessed for RETUNE eligibility by an in-built algorithm based on the routine data from the current visit. If eligible, the physician is prompted to hit a randomization button. If allocated to the intervention, the physician offers the intervention and collects consent of accepting participants (see supplement 2). If the person is allocated to the control group, the physician continues the visit without offering the intervention.

People who smoke more than one cigarette per day, who are 18 years or older, and who have signed the cohort and randomization consent are eligible. Pregnant women and people who use e-cigarettes, nicotine pouches, or nicotine patches at trial start are excluded.

### Randomization and blinding

For the randomization, we use a python-based stochastic treatment allocation algorithm based on the variance method to minimize imbalances simultaneously for region (French vs German speaking part of Switzerland), men having sex with men (yes/no), current drug user (yes/no), and number of cigarettes smoked per day [44]. A probability of 80% was set to assign the preferred treatment and avoid deterministic allocation. These key variables were selected based on routine cohort data availability, prior evidence from the literature indicating association to our outcome, and expert knowledge. The random allocation sequence is generated centrally at the SHCS Data Center, and the randomization module is integrated in the SHCS data collection tool. The SHCS physicians who perform the randomization have no access to the random allocation sequence and cannot see the allocation probabilities prior to assignment ensuring allocation concealment. The physicians who offer the intervention and collect the outcomes are aware of the group assignment. Participants in the control group are not aware of the trial (as per TwiCs design). The data analyst who manages the data and runs the analysis is aware of the group allocation.

### Trial intervention and comparator

The intervention consists of *offering* a menu of different nicotine substitute products. Available are e-cigarettes and e-liquids (menthol and tobacco flavor; nicotine concentrations 0 mg/ml, 3 mg/ml, 6 mg/ml, 12 mg/ml, 16 mg/ml), nicotine pouches (menthol and mountain herbs flavor), and nicotine patches (nicotine concentration 21 mg/24h, 14 mg/24h, 7 mg/24h). As e-cigarettes, we use the Aspire© pod system OBY with liquids from Gaiatrend©; the nicotine pouches are from Edelsnus© and the nicotine patches are from Nicotinell©. The participant can choose one of the products during the inclusion visit and have the opportunity to switch the product after 8 and 16 weeks. All products are provided free of charge for 24 weeks. Lost or defective products will be replaced. The participants receive an information brochure, with usage instructions, potential side effects, and how to request supply. Participants can discontinue product use in case of unacceptable side effects at any time. Standard smoking cessation counselling as currently done in the SHCS is permitted, including referral to special consultations as per smokers’ request. In the control group, only standard smoking cessation counselling is offered. Of note, no interventional products from the trial are dispensed to the control group. Likewise, participants are not directed to use e-cigarettes or nicotine pouches at their own costs. Standardization of the control group with a rigid protocol is not intended. After the intervention period, the participants remain in the SHCS receiving routine medical care and have access to usual smoking cessation services.

### Outcomes

The primary outcome was chosen based on smoking cessation core outcome sets [45, 46], a previous smoking cessation trial [33], discussions with the Participant Advisory Board, and the availability of smoking variables in the routinely collected data of the SHCS. The primary outcome is the proportion of people reporting tobacco cigarette use (yes/no), based on self-reported abstinence over the past 7 days at the 6-month visit (window 120–270 days) (see supplement 3). Secondary outcomes will generate evidence on long-term smoking cessation rates and broader health outcomes (Table 2).

**Table 2:** Primary and secondary endpoints

Primary endpoint	● Tobacco smoking status (yes/no) measured as self-reported abstinence in the last 7 day at 6-month visit (window, 120–270 days)
Secondary endpoints	● Tobacco smoking status (yes/no) measured as self-reported abstinence in the last 7 day at 12-month visit (window, 271–450 days) and 24-month visit (window, 630–810 days) ● Self-reported number of tobacco-based cigarettes smoked per day at 6, 12, and 24 months ● Self-reported use of any nicotine containing product other than tobacco cigarettes (yes/no) at 6 (window, 120–270 days), 12 (window, 271–450 days) and 24 (window, 630–810 days) months. If yes, self-reported use of e-cigarettes (yes/no) or nicotine pouches (yes/no) or patches (yes/no) or other (yes/no) ● Self-reported abstinence of any nicotine containing product (yes/no) at 12 (window, 271–450 days) and 24 (window, 630–810 days) months ● High Density Lipoprotein (HDL) and Low Density Lipoprotein (LDL) Cholesterol (mmol/l) at 6, 12, and 24 months ● Systolic and diastolic blood pressure (mmHg) at 6,12, and 24 months ● Body weight (kg) at 6, 12 and 24 months ● SCORE2-based [47] cardiovascular risk at 6, 12, and 24 months ● Occurrence of cardiovascular events (myocardial infarction, coronary angioplasty/stenting, coronary artery by-pass grafting, carotid endarterectomy, stroke, deep vein thrombosis, pulmonary embolism, heart transplantation) at 6, 12, and 24 months

### Measurements

Baseline data are collected by the treating physician as part of the routine visit. If randomized to the intervention, the physician records the participants’ product choice on a separate but linked REDCap-based data entry form. All trial endpoints (including laboratory values from routine measurements) and serious adverse events are extracted from the SHCS routine data [36].

To monitor the use and supply of the intervention products, the RETUNE study team contacts all intervention participants who chose an intervention product at weeks 8, 16, and 24. These follow-ups are performed via email or phone. At 8 weeks, the participants are asked if they used the product so far, but no product-specific adherence counselling is offered. At weeks 8 and 16, participants are asked about their desire to switch or continue the products. Adherence is not systematically assessed during or after the trial. At 24 weeks, they are asked about any potential adverse events of special interest, including new or increased nausea, emesis, headache, dizziness, respiratory symptoms, gingival pain, gingival bleeding, mouth ulcers, eczema, allergic skin reactions, mouth and tongue irritation, or change in the oral mucosa. Serious adverse events are collected as part of the routine SHCS data collection, extracted by the onsite staff or a trained member of the RETUNE study team, and evaluated regarding causality and severity by an independent physician. The sponsor investigator reports serious adverse events to the ethics committee within 15 days after becoming known to the sponsor investigator. An overview of the data collection is provided in Table 3. All data are stored for a minimum of 20 years.

**Table 3:**
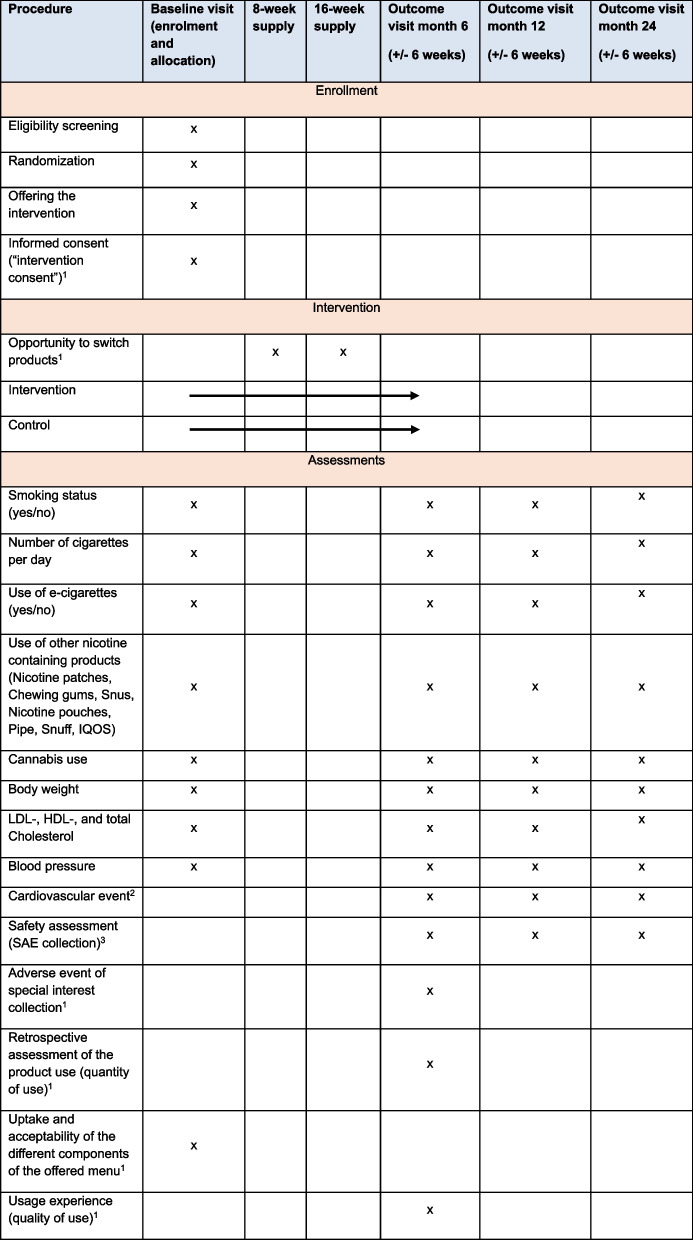
SPIRIT figure

^1^These data are only collected in the intervention group.

^2^Myocardial infarction, coronary angioplasty/stenting, coronary artery by-pass grafting, carotid endarterectomy, stroke, deep vein thrombosis, pulmonary embolism, heart transplantation

^3^A serious adverse event is any untoward medical occurrence that results in death or is life-threatening, requires in-patient hospitalization or prolongation of existing hospitalization, results in persistent or significant disability or incapacity, or causes a congenital anomaly or birth defect

### Sample size

**Fig. 2:**
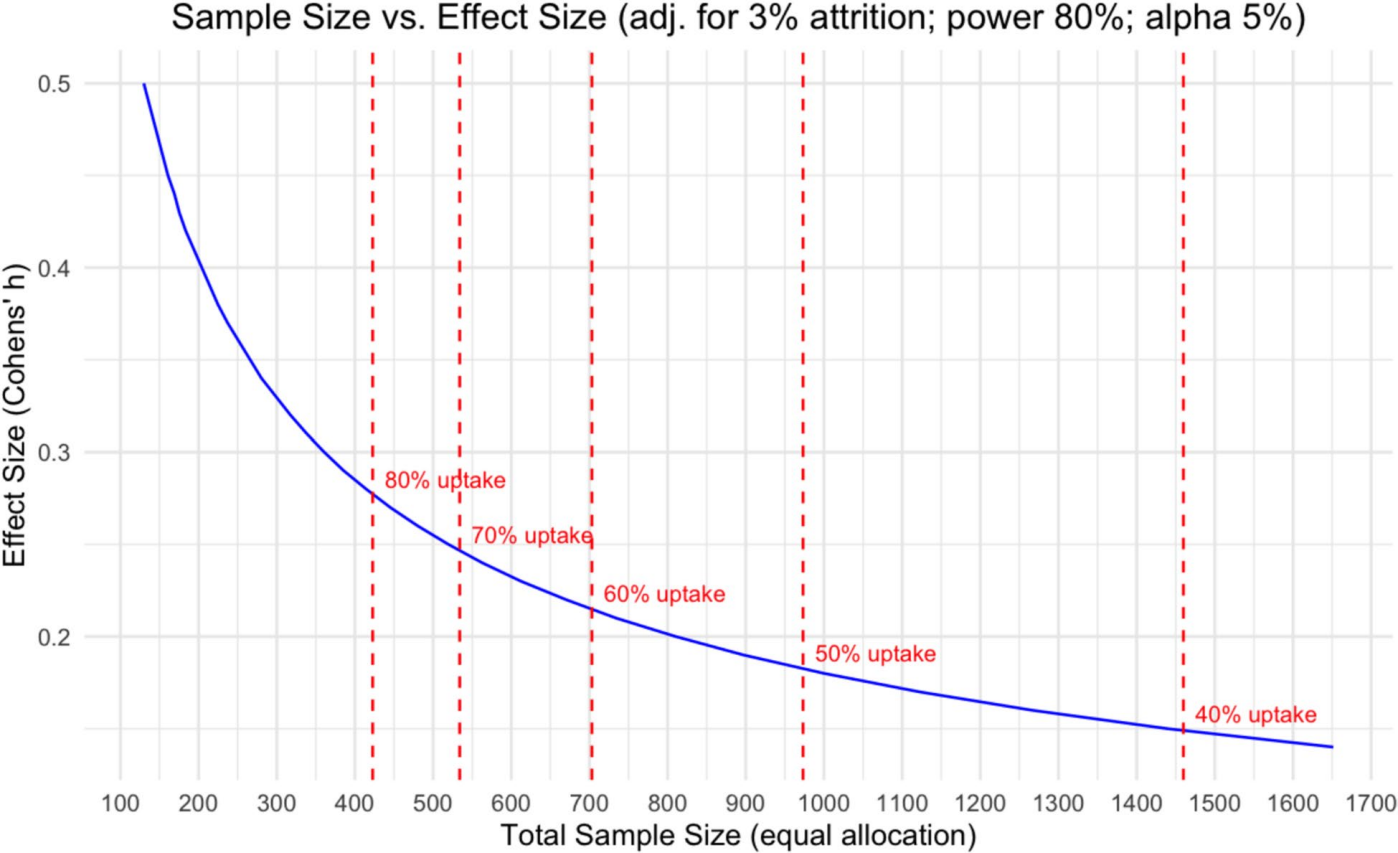
Influence of effect size on target sample size. The effect size is influenced by the uptake of the offered intervention. If the uptake is lower, then the effect size becomes exponentially smaller, influencing the target sample size. For our calculation, we used a two-sided alpha of 5%, a power of 80%, and an attrition rate of 3%. A Cohen’s effect size of 0.18 corresponds to a cessation rate of 8.5% in the control group and a diluted intervention effect of 14.25% (instead of 20%) in the intervention group, accounting for an uptake of 50% in the intervention group. The full R code for the sample size calculation is available on GitHub (https://github.com/alainamstutz/TwiCs_samplesize/blob/main/TwiCs_samplesize.md)

We assume a smoking cessation rate of 8.5% in the control arm (based on current SHCS data), a 20% cessation rate in the intervention arm (based on external evidence of similar trials [24–26]), and an attrition rate of 3% (based on SHCS data) [36]. Anticipating a low uptake of 50% in the intervention and thus dilution of the intervention effect, we aim at enrolling a total of 972 participants (486 in each arm) to achieve 80% power with a two-sided alpha level of 5%. End of 2022, the SHCS had about 1600 smokers treated in a RETUNE center. Following recommendations from the literature and approaches used in other TwiCs, we will reassess our uptake assumption at pre-specified timepoints (after 100, 200, and 300 randomized participants) [48, 49]. If the uptake is higher than expected, we will reduce the target total sample size (Fig. 2). We will not analyze outcomes at the sample size re-evaluation time points; thus, these do not constitute formal interim analyses with alpha spending, and no correction for multiplicity is required. If uptake falls below 45%, we will consider adding more sites or discontinuing the trial, depending on available budget and operational resources.

### Statistical analysis

The primary outcome will be assessed in the intention-to-treat set using a logistic regression model adjusted for the variables used for minimization (region [French vs. German speaking part of Switzerland], men having sex with men [yes/no], current drug user [yes/no], number of cigarettes per day) and will be reported as an adjusted odds ratio with 95% Wald confidence interval (CI) [50–52]. Superiority of the intervention versus control will be assessed according to the two-tailed *p*-value of the intervention regression coefficient and a significance level set to 0.05. Additionally, absolute risk difference and proportions of smoking cessation under intervention and control will be estimated using marginal standardization, with 95% percentile interval calculated from 1000 bootstrap samples.

Hypothesis testing will only be conducted for primary. Missing outcome data will be imputed using multiple imputation by chained equation techniques. Additionally, we will perform a sensitivity analysis where participants with missing outcomes will be excluded from the analysis (complete case analysis). For the secondary outcomes, we will use the same model as for the primary outcome but use linear regression in case of continuous outcomes and add baseline adjustment of the outcome where appropriate. The same strategy for missing outcome data will be applied to secondary outcomes. The secondary outcomes will be reported with 95% CIs to support descriptive interpretation and are of exploratory nature; no multiplicity adjustment is planned. We plan exploratory subgroup analysis (no pre-specified hypothesis) for the baseline number of cigarettes smoked per day (continuous), age (continuous), and sex (binary). We will include corresponding interaction terms—one at a time—in the adjusted logistic regression model. If a *p*-value of an interaction term turns out to be smaller than 0.1, we will assess the credibility of the effect using the ICEMAN tool [53]. Further details on subgroup, sensitivity, exploratory analyses, and the handling of missing data will be outlined in a statistical analysis plan that will be uploaded to the study repository prior to the database lock and data analysis.

### Internal pilot

The first 30 participants who accept the offered intervention (any smoking cessation product) form the internal pilot cohort. All 30 participants are contacted by phone 30 days after inclusion. We inquire about the experience with the product (flavor of liquids and nicotine pouches, nicotine concentration, handling) and on potential side effects. We also ask the participants if they wish to have other product options in the menu.

The pilot study investigates logistical challenges, generates knowledge about the popularity of the different components of the intervention, and explores safety issues. Based on the pilot results, especially the uptake of the different smoking cessation products and their flavors, we tailor our supply.

### Data management and monitoring

**Fig. 3:**
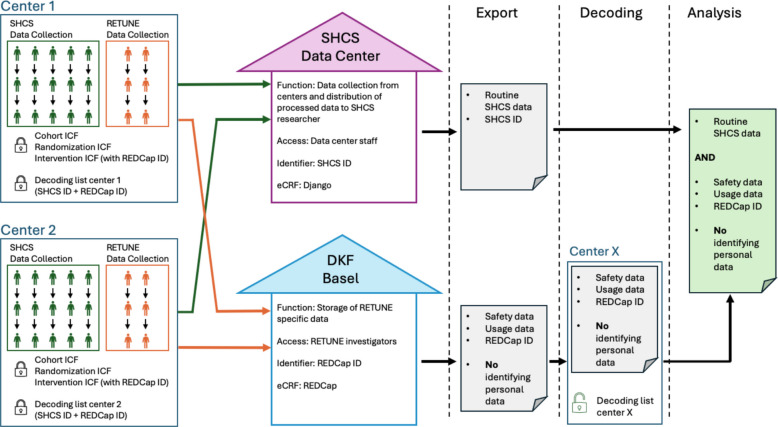
Data collection, processing, export, and decoding. Routine SHCs data is collected at the different SHCS centers via the SHCS data collection tool Django and stored and processed centrally at the SHCS data center at the University of Zurich. Data additionally collected for RETUNE (including identifying personal data like phone number, Email address or postal address) is collected via REDCap and stored at the Department of Clinical Research (DKF) at the University Hospital Basel. The decoding list which allows combining RETUNE data with SHCS data remains at the respective SHCS center. After completion of the trial, the RETUNE specific data is exported without identifying personal data. The decoding lists are used to combine the RETUNE data with the SHCS data at the respective centers. DKF Department Clinical Research, ICF Informed Consent Form, ID Identifier, RETUNE Reduce Tobacco Use In People Living With HIV, SHCS = Swiss HIV Cohort Study

RETUNE uses routinely collected data from the SHCS. The SHCS data collection tool is a Django-based web application hosted at the University of Zürich (https://www.djangoproject.com). We collect additional data only from participants in the intervention arm. This includes collecting adverse events of special interest among participants using the products and contact information of the participants to provide supply of the products. This data is recorded using password-protected REDCap (https://www.project-redcap.org/) surveys, hosted on secured servers of the University Hospital Basel. To secure participant confidentiality, only members of principal investigators and trained members of the core study group have access to this data. Before data export for the analysis, all identifying information is removed from the data set. Figure 3 provides an overview of the data flow in RETUNE and the requirements for different access and data protection levels. Data monitoring is conducted centrally and risk based. The central data monitoring team checks for consistency and plausibility of the entered data, e.g., between REDCap and the routine data. Inconsistency is resolved by the respective on-site routine SHCS personnel via email or phone. Due to the pragmatic and low-risk nature of the trial, no periodic audits are planned. RETUNE has no data monitoring committee. The principal investigators are responsible for the safety oversight. No safety stopping rules are predefined. In case of product-related harm, the trial participants are covered by the insurance of the sponsor institution (University Hospital Basel).

## Discussion

RETUNE aims to assess the effectiveness of offering a menu of different nicotine substitute products including e-cigarettes, nicotine pouches, and nicotine patches to tobacco smokers, regardless of their willingness to quit smoking. The TwiCs design facilitates the implementation of the “opt-out” approach including a menu of options for smoking cessation in a routine setting such as the nationwide SHCS [54].

The SHCS is an established, large-scale cohort with an excellent data structure, which is essential to use cohort data to check trial eligibility and assess trial outcomes. Its substantial size, coupled with the implementation of a cohort-wide randomization consent, enables the recruitment of sufficient participants for the RETUNE trial [55]. RETUNE will be the first trial using the TwiCs design in Switzerland which has the potential to be more cost and resource effective than traditional randomized trials by using established infrastructure for screening, recruitment, intervention delivery, and data collection [48]. It will generate high-quality, randomized evidence for an at-risk population of smoking PLWH. The TwiCs design is ideal for studying the “opt-out” approach and evaluating promising nicotine substitute products. Because the participants will remain in the SHCS after trial completion, we will be able to provide long-term follow-up data on smoking cessation—a frequent shortcoming of traditional smoking cessation trials. Finally, this trial is designed and conducted without any influence from tobacco companies or other commercial stakeholders.

RETUNE has the following limitations. First, due to the TwiCs design, we can only use routine cohort data as trial outcomes. Therefore, the primary endpoint (smoking cessation) will be patient-reported and not biochemically verified. Moreover, participants in the intervention group are aware of their group allocation. However, results from large-scale randomized trials indicate that estimates from 7-day self-reported abstinence rates are similar to biochemically verified abstinence rates [25, 27, 28]. Additionally, most trial participants will be part of the SHCS for many years, regularly answering routine cohort questions (including the question about smoking), and thus we expect the fully embedded trial to have a minimal effect on how participants report their smoking behavior. Also, participants in the control group have no stimulus for incorrect reporting since they are not aware of the trial. A second limitation arises from potential contamination effects by physicians who offer the intervention unintentionally to control group participants. Adequate training of trial personnel and monitoring of administered products will mitigate this issue. Third, the use of routine cohort data may lead to missing trial outcomes. However, the SHCS has a high data quality and completeness and since the cohort physicians are aware of the ongoing RETUNE trial, we anticipate few missing data, and most of them missing at random. Fourth, anticipating potential non-uptake of the offered intervention is a key challenge in planning TwiCs [54]. We used a pessimistic scenario of 50% uptake for our sample calculation. This seems feasible after discussion with physicians and patient representatives, and in comparison to previous trials [33]. Finally, we will reevaluate our assumption to ensure the feasibility of the trial.

RETUNE will provide randomized evidence on the effectiveness of offering a menu of different nicotine substitute products to smokers using an “opt-out” approach. Moreover, it adds valuable insights for smoking PLWH, a particular at-risk population, and efficiently embeds the trial in a nationwide cohort through the TwiCs design.

Protocol version: 1.2 (dated 08.08.2025). Previous versions: 1.1 (dated 06.01.2025; addition of new centers and clarification of secondary endpoints), 1.0 (dated 28.11.2024; addition of new centers, clarification of inclusion criteria, and addition of monitoring plan).

### Trial status

Start of recruitment: February 20th, 2025. The end of recruitment is estimated to be in January 2027.

## Supplementary Information

Additional file 1: Intervention consent form

Additional file 2: Primary outcome question

Additional file 3: SPIRIT checklist 

## Data Availability

The product information brochures are available on request or on the RETUNE website (retune-trial.com). The full trial protocol is available on the trial registry (NCT06789692), on the RETUNE website (retune-trial.com), or after request. The R code for the sample size calculation is available on GitHub (https://github.com/alainamstutz/TwiCs_samplesize/blob/main/TwiCs_samplesize.md). The trial analysis code will be made available on GitHub. The data will not be publicly available as per SHCS regulations, but an anonymized analysis dataset will be available upon request. Investigators with a data request should send a proposal to the SHCS which will be evaluated by the Scientific Board of the SHCS. The process follows the regulations from the SHCS described on the website: https://www.shcs.ch/for-researchers/open-data-statement-shcs/

## Abbreviations

CI: Confidence interval
E-cigarettes: Electronic cigarettes
FDA: US Food and Drug Administration
PLWH: People living with HIV
RETUNE: Reduce Tobacco Use iN pEople living with HIV in Switzerland
SHCS: Swiss HIV Cohort Study
TwiCs: Trials within Cohorts
