# Supplementary material for: Offer of a menu of different nicotine substitute products to REduce Tobacco Use iN pEople living with HIV (RETUNE): a protocol for a pragmatic randomized trial within the Swiss HIV Cohort Study
Source: Trials. 2026 Apr 8;27:372. doi: 10.1186/s13063-026-09622-6 (PMC13181873; doi:10.1186/s13063-026-09622-6)
Supplement: Supplementary file 1 — Additional file 1: Intervention consent form [file 13063_2026_9622_MOESM1_ESM.pdf]

Offer of a menu of different nicotine substitution products (e-cigarettes, nicotine pouches and nicotine patches) to quit smoking tobacco cigarettes – The RETUNE study

Why are we conducting this study?

- We want to find out if less harmful cigarette alternatives such as e-cigarettes, nicotine pouches, and nicotine patches are effective to quit smoking.

What happens if I take part?

- You are offered three products that can help you stop smoking tobacco cigarettes:
  - **E-cigarettes**
  - **Nicotine pouches**
  - **Nicotine patches**
- You can choose one of the products to take home and try out. You will receive instructions and a brief explanation of how to use it.
- After 6 weeks, we will contact you to ask how it is going and to provide you with new supply of the product if you wish so. In total, you will get the chosen product free of charge for 6 months.
- No additional data, examinations or blood are needed. All data is encrypted and safely handled as all data collected in the cohort.

What are the risks and benefits associated with these products?

Benefits

- These alternative nicotine products are <u>less harmful</u> than conventional cigarettes and might help you quit smoking tobacco cigarettes.
- You will receive your desired alternative nicotine products free of charge for 6 months.
- You will contribute to a better understanding of newer nicotine replacement products.

Risks and strain on the body

- Albeit less than conventional tobacco cigarettes, e-cigarettes, nicotine pouches, and nicotine patches may have their own health risks such as local (mucosa) skin irritation, and allergic or systemic reactions such as nausea and headache. E-cigarettes can have toxic lung effects causing pneumonia-like manifestations.
- Long-term risks of these products have not been conclusively examined.
- Use of multiple nicotine containing products at the same time should be avoided.

By signing the declaration at the end of this document, you are indicating that you have understood the contents of this document.

Detailed information

1. Objective and selection

In this study we investigate the efficacy of a menu of different nicotine substitutional products terms of quitting smoking after 6 months. The menu contains of e-cigarettes, nicotine pouches, and nicotine patches. The study includes tobacco smoking people living with HIV in the Swiss HIV Cohort Study.

We ask you because you are a participant of the Swiss HIV Cohort Study, are 18 years or older, have signed the randomization consent, and smoke one or more tobacco cigarettes per day.

2. General information

Tobacco smoking is a major risk factor for lung cancer and cardiovascular diseases such as heart attacks. Quitting smoking is important but difficult. New approaches to quit smoking focus on harm reduction by replacing tobacco cigarettes with less harmful alternatives.

- **E-cigarettes** imitate tobacco cigarettes. They heat up nicotine containing liquids to inhale nicotine in form of vapor in contrast to smoke like in traditional tobacco cigarettes. By avoiding burning tobacco, e-cigarettes produce a significantly lower amount of harmful chemicals than tobacco cigarettes and can reduce exposure to cancer causing toxins. E-cigarettes come close to the smoking routine and mimic experiences of smoking, such as the feeling of holding a cigarette, or inhaling smoke into the throat ("throat hit") or exhaling the cloud of smoke. In this study, we will use the Aspire© pod system OBY e-cigarette. The device is authorized and certified for sale in Switzerland.
- **Tobacco-free nicotine pouches** are nicotine products in a pouch, that are clamped under the lip and provide nicotine through the oral mucosa. In contrast to snus, nicotine pouches do not contain tobacco. Studies suggest that nicotine pouches are safe and well tolerated in healthy participants. We will use nicotine pouches from the brand Edelsnus©. The product is authorized and certified for sale in Switzerland.
- **Nicotine-containing patches** are a widely used form of nicotine replacement therapy and an established standard of care against withdrawal symptoms. Nicotine uptake through the skin is slow and nicotine patches need about one hour to reach the maximum nicotine concentration. Afterwards, they create a steady nicotine level for up to 24 hours. We will use patches from the brand Nicotinell©. These patches are classified as a drug, have been approved by Swissmedic and have been used in clinical practice for many years.

We are carrying out this study in accordance with Swiss law and in compliance with internationally recognized guidelines. The study has been reviewed and authorized by the relevant ethics committee.

More information about the study is available via clinicaltrials.gov (link and registry number to follow).

3. Procedure

1. Your clinician offers you a preference-based smoking substitution menu. You choose one product to test as an alternative to tobacco smoking.
2. The products are handed out directly after the consultation and are free of charge for 6 months.
3. To guarantee the supply of the interventional products and to conduct surveys on possible side-effects and usage we ask you to provide us with your postal address and preferred communication channel (telephone number, email address).
4. After 6 weeks, we will contact you to renew your supply of the products.

The data will all be collected during regular cohort visits, which take place every 6 months. No additional visits or blood samples are necessary. You will continue to receive the same medical care within the Swiss HIV Cohort Study. The study is being conducted at the University Hospital Basel, University Hospital Zurich,

Inselspital Bern, Cantonal Hospital St. Gallen, University Hospital Lausanne and the University Hospital Geneva.

4. Benefits

The intervention is designed to help you quit smoking tobacco cigarettes. Given the major health burden caused by tobacco smoking, especially in people living with HIV, the possible benefits may outweigh the possible risks (see 6. below). You benefit by reducing your risk of cardiovascular diseases and cancer caused by smoking tobacco. Similarly, society and smokers in other settings will profit from improved knowledge about smoking cessation.

5. Voluntary use of offered products

You can decide to stop using the product at any time. In this case, you can dispose the products. Your medical care and treatment remain unchanged regardless of whether you choose a product or not, and you will still participate in the Swiss HIV Cohort Study with scientific use of your data.

6. Risks and strains on the body

This is a low-risk study, and the application of the different products should reflect real-life. All products used in this study are approved by the Swiss authorities and freely available on the market. The products used in this study are compatible with European Union regulations or, in the case of the nicotine patches, approved by Swissmedic. All products can cause local reactions and irritation of the skin or mucosa. All products can lead to systemic reactions of the nicotine intake such as nausea and headache. All products can cause allergic reactions. If you have a reaction of this type or any other side-effects, you should discontinue the products and contact your treating physician. The products should not be seen as a healthy alternative, but as a less harmful alternative to tobacco smoking. You should not use one of the products and tobacco cigarettes at the same time.

For women who are pregnant or of childbearing potential

If you are pregnant, you cannot participate in this study. Stopping smoking during pregnancy is strongly recommended. The treating physician will discuss the further procedure with you.

7. Results

The Swiss HIV Cohort Study will publish a summary of the overall results at the end of the study (anticipated beginning 2027) as usual in their newsletter and other communication channels.

8. Data and sample confidentiality

8.1 Data processing and encryption

All relevant data are collected within the routine data collection from the cohort. Your data is encrypted in accordance with the regulations of the Swiss HIV Cohort Study. No additional blood samples or visits are conducted as part of this study.
However, to organize your supply of products and ask you about how it is going with the product, we would like to contact you after 6 weeks. For this reason, we ask for your contact data (postal address, email address, phone number). This data, including your name, will be recorded in a secured password-protected study database at the University Hospital Basel, separate from the Swiss HIV Cohort Study database. Only the RETUNE study team (see below) and your treating physicians and nurses will have access to this data.

8.2 Data protection and safeguarding of samples

All Swiss laws and guidelines from the Swiss HIV Cohort Study pertaining to data protection will be strictly kept to. Your data will be analyzed and shared in anonymous format for scientific publication (i.e. without your name, contact info, etc.). Anonymized data might be shared with other researchers according to the Swiss HIV Cohort Study data sharing laws. No samples are collected for this study.

8.3 Inspection rights in the event of audits

This study may be audited by the relevant ethics committee. In this case, the investigator is obliged to make your date accessible for auditing. All parties involved must maintain strict confidentiality.

9 Remuneration

You will receive the products free of charge for 6 months. You will not receive any remuneration for taking part in this study.

10 Liability

In the event of study-related damage or injuries, the liability of the University Hospital Basel provides compensation.

11 Financing

This study is funded by the Swiss National Science Foundation, the Novartis Foundation for Medical-Biological Research and the Swiss Tobacco Prevention Fund (Bundesamt für Gesundheit).

12 Contact persons

If you have any questions regarding the study or regarding the products, please contact:

Dr. Example
Example clinic
Example address
Example phone number
Example email

For urgent medical problems, please contact your treating physician.

13 RETUNE Study team

Christof Schönenberger, MD	(co-principal investigator)
Alain Amstutz, MD PhD	(co-principal investigator)
Prof. Matthias Briel, MD PhD	(co-principal investigator)

Written declaration of consent for participation in a clinical study
Please ask if you do not understand something or would like to know anything else.

**BASEC-number:**	2024-02417
**REDCap Identifier**	
**SHCS Identifier**	
**Title of the study**	RETUNE: REduce Tobacco Use iN pEople living with HIV in Switzerland<br><br>Offer of a menu of different nicotine substitution products (e-cigarettes, nicotine pouches and nicotine patches) to quit smoking tobacco cigarettes.
**Responsible institution**	University Hospital Basel<br>Division of Clinical Epidemiology<br>Totengässlein 3, 4051 Basel
**Location of the study**:	
**Investigator on site:**	
**Participant:**<br>Surname and first name in block capitals:	

- I have been informed of the purpose and procedure of the study, possible advantages and disadvantages and any possible risks both verbally and in writing by the undersigned investigator.
- My questions concerning participation in this study have been answered. My participation is voluntary. I will retain the written information and keep a copy of my written declaration of consent.
- I can stop the participation in the RETUNE study at any time without giving reasons.
- I consent to share my postal address for the supply of the chosen product, and email or phone number to contact me.

Place, Date	Participant's signature

Confirmation by the investigator: I hereby confirm that I have explained the nature, significance and implications of the study to this participant.

Place, Date	Investigator's surname and first name in block capitals<br><br>Investigator's signature
