# Supplementary material for: Offer of a menu of different nicotine substitute products to REduce Tobacco Use iN pEople living with HIV (RETUNE): a protocol for a pragmatic randomized trial within the Swiss HIV Cohort Study
Source: Trials. 2026 Apr 8;27:372. doi: 10.1186/s13063-026-09622-6 (PMC13181873; doi:10.1186/s13063-026-09622-6)
Supplement: Supplementary file 2 — Additional file 2: Primary outcome question [file 13063_2026_9622_MOESM2_ESM.docx]

**Supplement 2**

**Appendix: Question to assess the primary outcome**

*«Does the patient smoke cigarettes?»*

0=no

1=yes

If no:

*«Did the patient smoke any cigarette in the last 7 days?»*

0=no

1=yes

Blank=missing

If yes:

*«How many cigarettes did the patient smoke per day?»*
